# MALDI mass spectrometry imaging workflow for the aquatic model organisms *Danio rerio* and *Daphnia magna*

**DOI:** 10.1038/s41598-022-09659-y

**Published:** 2022-05-04

**Authors:** Elisabeth Schirmer, Sven Ritschar, Matthias Ochs, Christian Laforsch, Stefan Schuster, Andreas Römpp

**Affiliations:** 1 Bioanalytical Sciences and Food Analysis, University of Bayreuth, Bayreuth, Germany; 2 Animal Ecology I and BayCEER, University of Bayreuth, Bayreuth, Germany; 3 Animal Physiology, University of Bayreuth, Bayreuth, Germany

**Keywords:** Electrophysiology, Mass spectrometry, Ecology, Environmental sciences, Analytical chemistry, Freshwater ecology

## Abstract

Lipids play various essential roles in the physiology of animals. They are also highly dependent on cellular metabolism or status. It is therefore crucial to understand to which extent animals can stabilize their lipid composition in the presence of external stressors, such as chemicals that are released into the environment. We developed a MALDI MS imaging workflow for two important aquatic model organisms, the zebrafish (*Danio rerio*) and water flea (*Daphnia magna*). Owing to the heterogeneous structure of these organisms, developing a suitable sample preparation workflow is a highly non-trivial but crucial part of this work and needs to be established first. Relevant parameters and practical considerations in order to preserve tissue structure and composition in tissue sections are discussed for each application. All measurements were based on high mass accuracy enabling reliable identification of imaged compounds. In zebrafish we demonstrate that a detailed mapping between histology and simultaneously determined lipid composition is possible at various scales, from extended structures such as the brain or gills down to subcellular structures such as a single axon in the central nervous system. For *D. magna* we present for the first time a MALDI MSI workflow, that demonstrably maintains tissue integrity during cryosectioning of non-preserved samples, and allows the mapping of lipids in the entire body and the brood chamber inside the carapace. In conclusion, the lipid signatures that we were able to detect with our method provide an ideal basis to analyze changes caused by pollutants in two key aquatic model organisms.

## Introduction

Pollution not only in marine but also in freshwater ecosystems rises due to an ever increasing quantity and diversity of chemicals that are released into the environment^1^. Of particular concern are lipophilic substances that affect the physiological homeostasis of aquatic organisms by interfering with their lipid composition^2^. Considering that lipids are crucial for tissue architecture and function^3^, it is not surprising that changes in lipid pattern are often correlated with pathological processes^4,5^. Hence, it is important to understand how chemical stressors affect the composition and spatial distribution of lipids in aquatic organisms. In this approach, knowing the lipid signature that is characteristic for a normal healthy tissue would be used as a reference^6,7^ against which pollutant-exposed tissue can be compared. Here, we will use matrix-assisted laser desorption/ionization (MALDI) mass spectrometry imaging (MSI), a molecular imaging technique that is capable of analyzing the molecular composition and spatial distribution of complex biological samples. It allows to identify compounds of interest and to visualize their distribution within thin tissue sections^8–10^. Identification in complex biological tissue typically requires high resolution mass spectrometry with high mass accuracy for reliable identification^11^. In MALDI MSI experiments, the sample is scanned pixel-wise by a laser and a mass spectrum is recorded at every pixel with the signals measured as *m/z* (mass-to-charge ratio). The generated image represents the intensity of a detected analyte against the respective x/y coordinate of each pixel. This way, a compound-specific distribution map^12^ is obtained that can be linked to anatomical features^8,13^. This approach has been highly successful for a wide range of compound classes including metabolites, drug compounds^14^, lipids^15^ and proteins^16^ in biological samples. We have recently shown that it even enables the characterization of anatomical features in sections of *Eisenia fetida*—a terrestrial model system for ecotoxicology studies—using MS imaging in combination with FTIR imaging^17^.

Zebrafish *Danio rerio* and the waterflea *Daphnia magna* are two widely used aquatic model organism in the field of environmental science and ecotoxicology^18–20^. The use of *D. rerio* represents an excellent compromise between complexity and practical simplicity and further often allows conclusions for both animal and human health^21,22^. In the present context, the small size of the fish offers the opportunity to investigate the lipid distribution in neuronal and non-neuronal compartments in one measurement. Furthermore, *D. rerio* offers the unique possibility to examine the lipid composition in a central command neuron and its axon. This can be individually identified from fish to fish and the availability of transgenic GFP-lines allows a simple selection process of material to be used for MALDI MSI. This neuron, the so-called Mauthner neuron, is the largest neuron in the vertebrate central nervous system^23^. Studies have already used the unique properties of this neuron to demonstrate the effect of environmental contaminants on key neuronal functions^24^.

The filter feeder *Daphnia* on the other hand is a key-stone species in the food web of nearly every lentic aquatic habitat, as it serves as a link between autotrophic algae and consumers of higher trophic levels, such as fish^25^. Due to its global distribution, easy cultivation and its clonal reproduction, the genus *Daphnia* is frequently used for research in environmental toxicology. Especially *D. magna* is a well-established organism for eco-toxicological testing^26^ and—similarly as *D. rerio*—is on the American National Institute of Health (NIH) to their list of model organisms for biomedical research^20^. *D. magna* provides a biological system with a high sensitivity for the detection of even weak effects to a wide range of pollutants on various levels reaching from life-history^27^ to physiology and molecular effects^28,29^. For *Daphnia*, it is already described, that the lipidome is impacted by environmental factors such as food quality^28^ or pollutants^30^. However, to date it is not known how environmental stressors affect composition and spatial distribution of lipids in different tissues.

Here, we use technological improvements in MALDI MSI to establish a sample preparation workflow for correlating lipid pattern with anatomical features of more extended structures (neuronal and non-neuronal) in both model organisms. For *D. rerio*, our method provides a detailed view of lipid composition across different tissues down to cellular resolution in a single neuron. In *D. magna* we show a way for ensuring tissue integrity during cryosectioning despite the calcified exoskeleton that has plagued earlier work^31^. This allowed us to visualize the lipid distribution within different tissues of *D. magna* by MALDI MSI. The observed lipid pattern in *D. magna* and *D. rerio* may serve as references for future studies on changes in lipid signatures after exposure to environmental pollutants.

## Results and discussion

### MALDI MSI sample preparation workflow for *D.rerio* and *D.magna*

**Figure 1:**
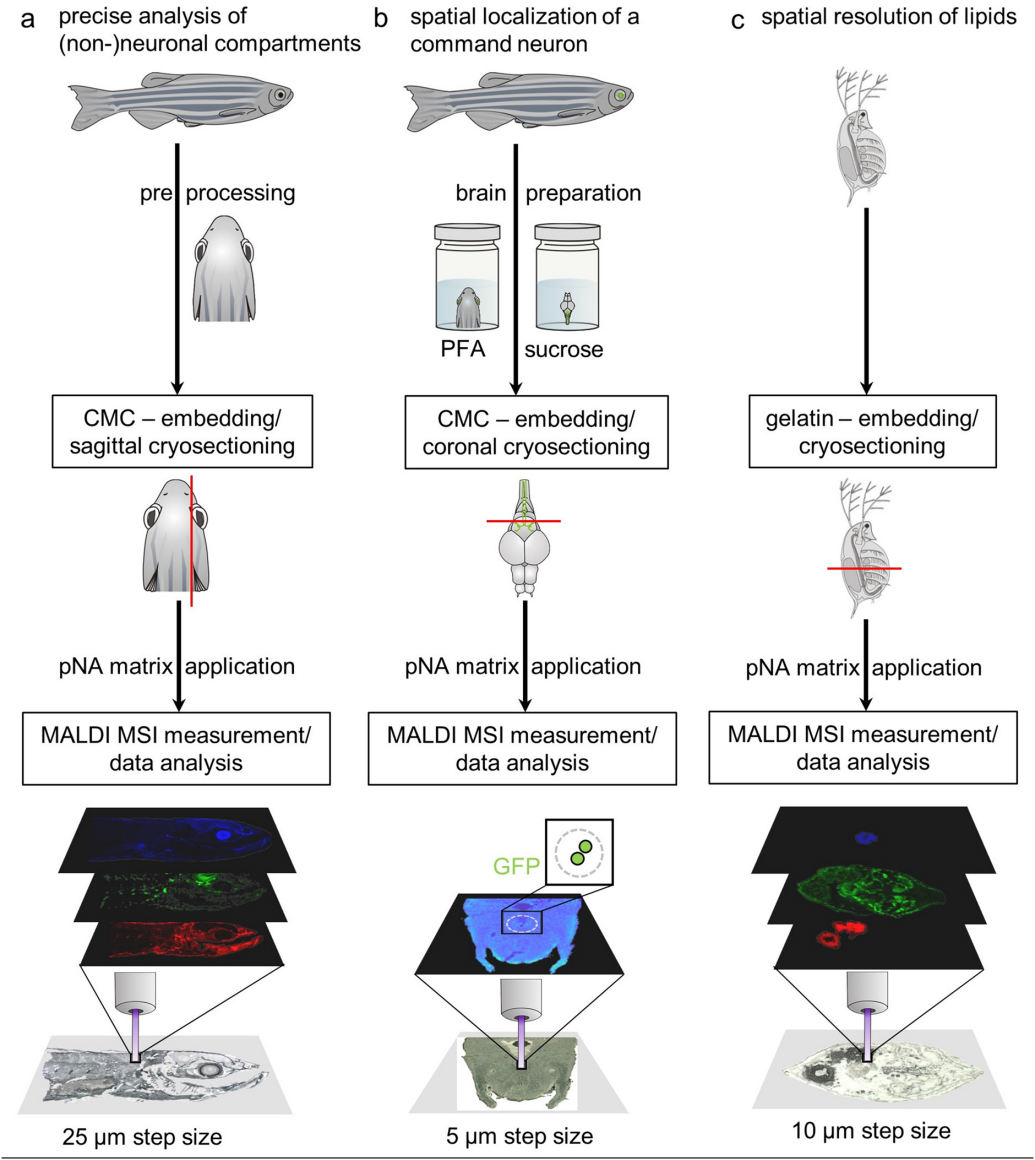
MALDI MSI workflow in *D. rerio* and *D. magna*. (**a**) Workflow for the precise analysis of lipid patterns visualizing anatomical features within neuronal and non-neuronal compartments. For sample preprocessing the adult zebrafish was cut cranial to the anal fin. The prepared sample was embedded in carboxymethylcellulose (CMC) and sagittal cryosections were prepared as indicated by the yellow line. Sections were covered with 4-Nitroaniline (pNA) matrix and MALDI MSI experiments were carried out in positive ion mode with 25 µm step size. (**b**) Workflow for the spatial localization of a command neuron in brain sections. Adult zebrafish were cut cranial to the anal fin and fixated in paraformaldehyde (PFA). After removal of the brain, it was fixated in sucrose and subsequently was embedded in CMC. Coronal cryosections were prepared as indicated by the red line. Brain sections (70 µm) were coated with pNA matrix and MALDI MSI measurements were carried out in positive ion mode with 5 µm step size. Prior to matrix application the sections were investigated by fluorescence microscopy to ensure the presence of the soma of the command neuron. (**c**) Workflow revealing the spatial distribution of lipids in whole body sections of *D. magna*. The sample was directly embedded in gelatin solution (8%) and sections (of 18 µm thickness) were prepared and coated with pNA matrix. MALDI MSI experiments (10 µm step size) were performed as described before.

MALDI MSI is a molecular imaging technique capable of identifying and visualizing the distribution of lipids within biological tissues^8^. The differentiation of anatomical features within different tissue types requires, obviously, that the samples are prepared and handled so as to not disturb composition and distribution within tissue sections that are to be analyzed. Therefore, developing a suitable sample preparation workflow is a highly non-trivial but crucial part of this work and needs to be established first. Figure 1 gives an overview of our procedures. Our work includes three different workflows optimized to obtain lipid patterns from sagittal sections (Fig. 1a), somatic and axonal regions of an identified central command neuron (Fig. 1b) in *D. rerio* and whole-body sections of *D. magna* (Fig. 1c). A few characteristic features are discussed in the respective subsections below and full experimental details are provided in the Methods section.

### Lipid characterization in sagittal sections of *D.rerio*

Figure 1a shows the MALDI MSI workflow for the analysis of neuronal and non-neuronal compartments in *D. rerio*. Of particular importance were tissues that would potentially be important for the uptake of environmental chemicals (e.g. gills, eye) or that could be particularly affected by them (e.g. brain, liver). Prior to sample preparation we used 2-phenoxyethanol for euthanization^32^. Tissue was embedded in 3% CMC (carboxymethyl cellulose) which is compatible with mass spectrometric analysis. MALDI MSI was performed on sagittal cryosections with a 25 µm step size.

**Figure 2:**
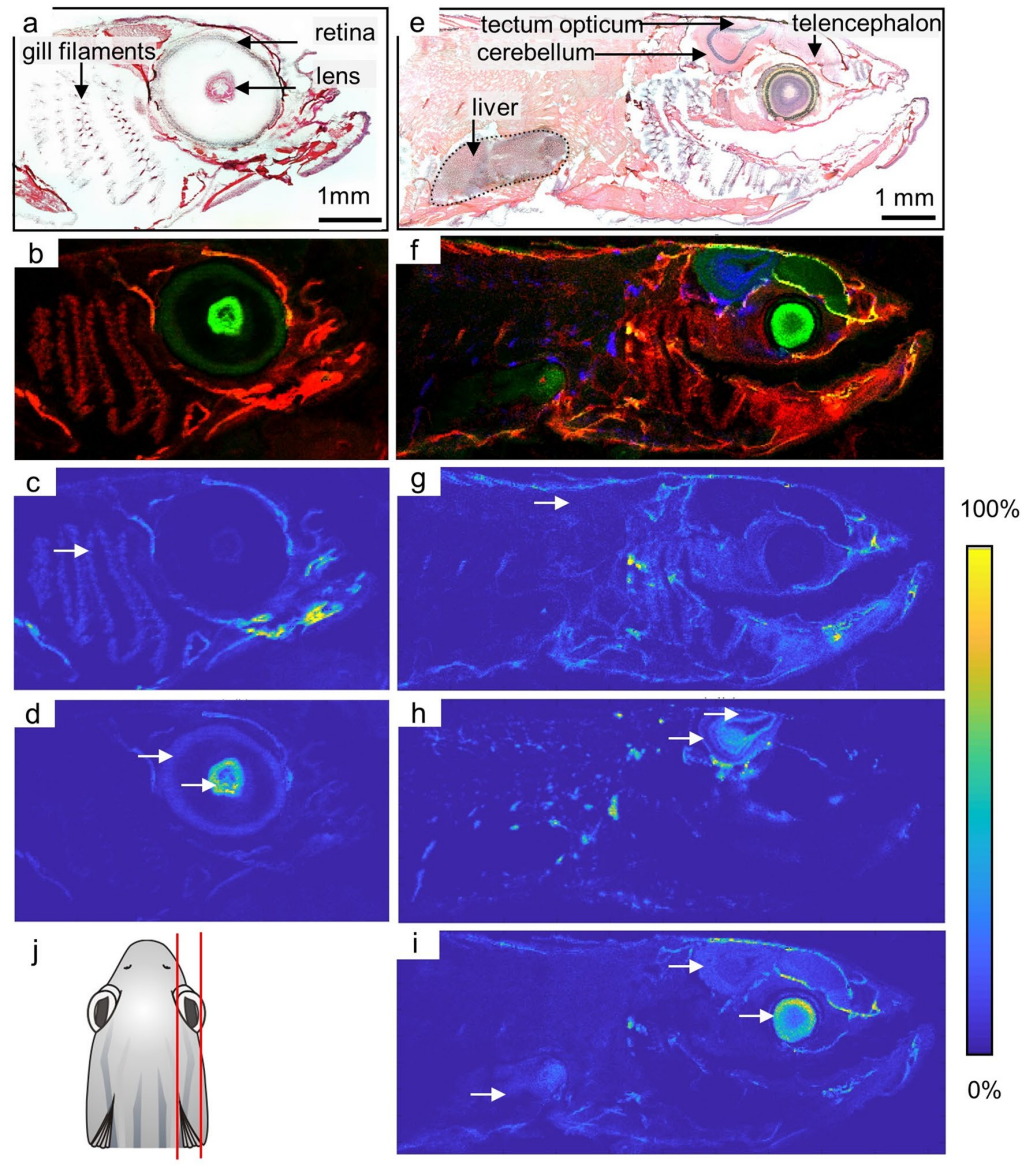
MALDI MS imaging of lipids in neuronal and non-neuronal compartments of *D. rerio*. (**a**,**e**) H&E (hematoxylin and eosin) staining of the sagittal cryosections in adult zebrafish showing detailed structures within the gills’ filaments, eye (retinae, lens), brain (*telencephalon, tectum opticum, cerebellum*) and liver. (**b**) RGB Overlay of PC (O-32:0) [M+H]^+^ (red) and PC (30:0) [M+K]^+^ (green). (**c**,**d**), (**g–i**) Positive ion mode MS images. (**c**) Ion image of PC (O-32:0) [M+H]^+^ showing high intensity in the region of the gills’ filaments (white arrow). (**d**) Ion image of PC (30:0) [M+K]^+^ visualizing the lens and the retina of the eye (white arrow). (**f**) RGB Overlay of PC (O-32:0) [M+H]^+^ (red colored), PC (O-36:1) [M+K]^+^ (blue colored) and PC (40:6) [M+H]^+^ (green colored). (**g**) Ion image of PC (O-32:0) [M+H]^+^ shows distribution with high intensity in non-neuronal tissue, exemplarily shown for muscle tissue (white arrow). (**h**) Ion image of PC (O-36:1) [M+K]^+^, illustrates the structural differentiation within the tectum opticum and parts of the cerebellum. (**i**) Ion image of PC (40:6) [M+H]^+^ shows distribution within the brain and eye and presents the liver as relative homogenous tissue (white arrows).

Results based on this workflow are shown in Fig. 2. The position of the sagittal sections is indicated in Fig. 2j. MS images were acquired at 25 µm pixel size (240 pixel × 142 pixel) in positive ion mode in the mass range of *m/z* 600–1000. Our workflow allowed us to visualize the retina and the lens due to the distribution of the phospholipid phosphatidylcholine (PC) (30:0) [M + K]^+^ (*m/z* 744.4940) (Fig. 2d). The RMSE for this signal in the entire MS image was 1.3 ppm (based on 26,056 spectra). This high mass accuracy was obtained for all MS imaging data in this study (see supplementary information Table S1 for more details) and is the basis for reliable identification of imaged compounds.

Lipid analysis of the eye (as a whole) using MSI was already performed by Liu et al.^2^, who aimed at analyzing the changes in lipid metabolism in zebrafish upon fipronil exposure. This work also maintained the tissue integrity and detected changes in the lipid composition of the eye. Our procedures extend these findings by allowing now to also differentiate between retina and lens. Thus, we now provide a more detailed picture that could differentiate which of the many different aspects of the eye is most affected.

The anatomical fine structure of the gills are visualized by the phospholipid PC (O-32:0) [M+H]^+^ (*m/z* 720.5902) shown in Fig. 2c. As the respiratory organs the gills are optimized for maximum diffusion between the surrounding water and blood. They are thus a major point of entry for many environmental contaminants. Accordingly, the uptake of pollutants via the gills is a central topic in ecotoxicology^33–36^ using either spatial (e.g. histological examinations^35^) or molecular (transcriptomics^33^) analyses. Our workflow has the advantage of combining both spatial and molecular information. Supporting the importance of such a combination, Perez et al.^36^ demonstrated that pollutants can accumulate within gill tissue of zebrafish. The authors analyzed AMMOENG 130, a toxic ionic liquid, which they localized both in gill and nervous tissue. The study focused on the distribution of the ionic liquid within whole body sections at 200 µm pixel size. In contrast, our workflow allows much higher spatial resolution (25 µm pixel size) and higher molecular specificity due to accurate mass measurements. A study by Stutts et al.^37^ analyzed sagittal sections of zebrafish with infrared matrix-assisted laser desorption electrospray ionization. They were able to obtain whole body sections and obtain high mass accuracy data at 100 µm pixel size. Our results do not have the advantage of covering the complete organisms in one slide, but have the advantage of higher spatial resolution (25 µm) and supposedly higher signal intensity which results in more details in the lipid imaging. Figure 2b illustrates this aspect showing an RGB overlay of both obtained lipid patterns, presenting structures of the eye and the gills, with PC (O-32:0) [M+H] + displayed in red and PC (30:0) [M+K] + displayed in green. The H&E stained section is shown for comparison in Fig. 2a.

The retina is a part of the central nervous system^38^. It is therefore fitting that the next sagittal section shows the lipid PC (40:6) [M+H]^+^ (*m/z* 834.6007) both within brain tissue and the retina (arrows in Fig. 2i). This lipid clearly allows the visualization of the *tectum opticum,* parts of the *cerebellum* and of the *telencephalon*. Further details of the *tectum opticum* are revealed by the distribution of PC (O-36:1) [M+K]^+^ (*m/z* 812.5930) given in Fig. 2h. The ability to separate the *tectum opticum* from other compartments is interesting, considering, for instance, the study of Strungaru et al.^39^, who used immunocytochemistry to analyze the impact of Deltamethrin on the *telencephalon*, *tectum opticum* and *cerebellum*. Our method now allows to analyze specific compounds associated with these major brain areas and would, for example, allow to analyze the effects seen by Mishra et al.^40^ of to the pesticide Chlorpyrifos on the *tectum opticum* on another freshwater teleost. In contrast to the neuronal region, non-neuronal tissue of this section could also be visualized by the distribution of PC (O-32:0) [M+H]^+^ (*m/z* 720.5902) in Fig. 2g. Additionally, the liver could be localized and the distribution of the lipid PC 40:6 [M+H]^+^ matches the homogenous consistence of this organ (Fig. 2i). The liver as a central organ involved in detoxification is also target of impact assessments of toxicants in zebrafish. For instance, Jia et al.^41^ analyzed the impact of fungicides (strobilurins) on the liver of *D. rerio* by deploying assays for the detection of antioxidant enzymes. The RGB overlay of distributions of PC (O-32:0) [M+H]^+^ displayed in red, PC (O-36:1) [M+K]^+^ displayed in blue and PC (40:6) [M+H]^+^ displayed in green matches the anatomical features observed in the H&E staining (Fig. 2f).

### Lipid characterization in a central command neuron in *D.rerio*

**Figure 3:**
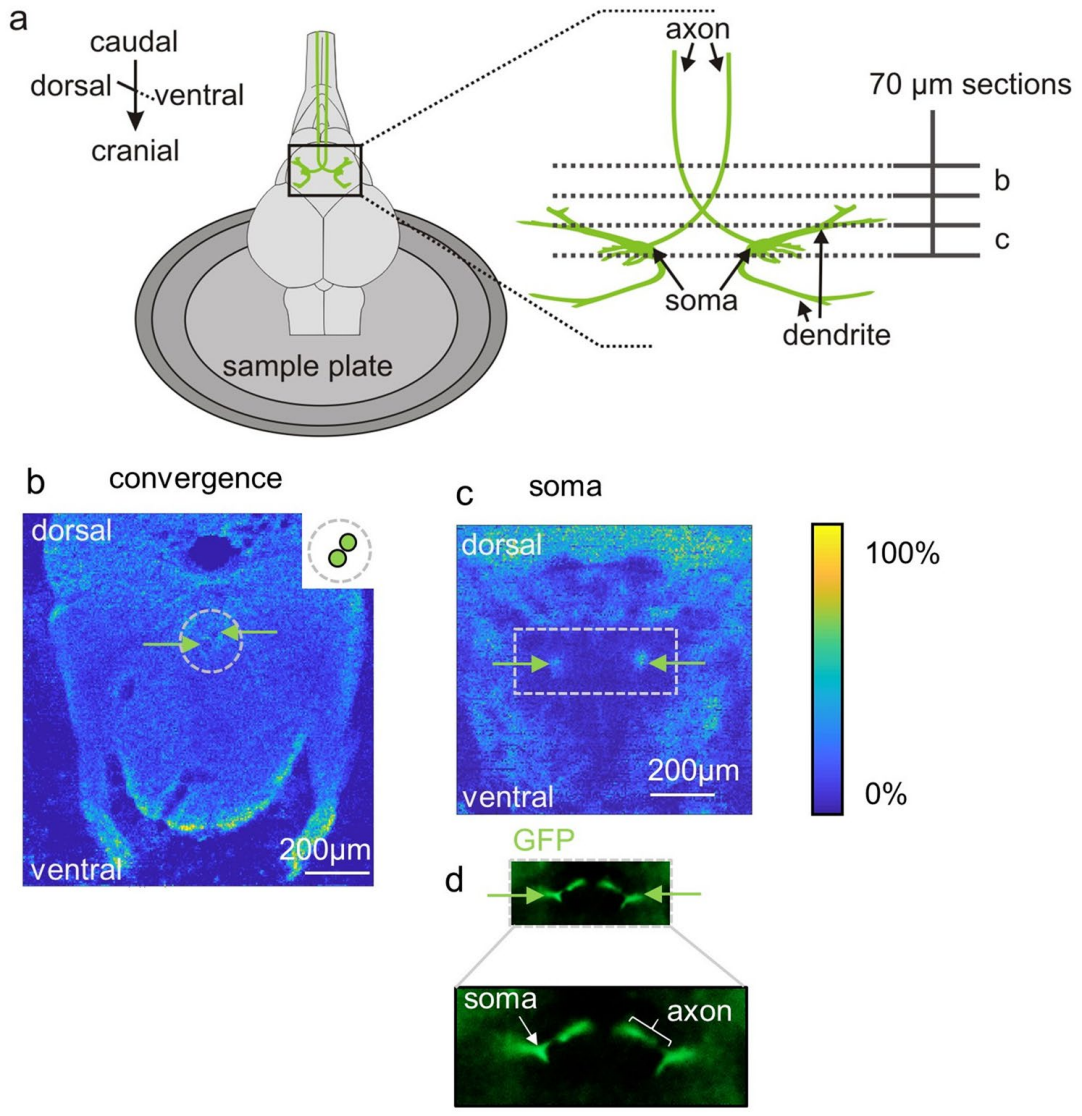
Spatial localization of a central command neuron within tissue sections of zebrafish using MALDI MSI. (**a**) Schematic representation of a zebrafish brain fixated on a sample holder, with the central command neurons (the pair of Mauthner neurons) shown in green. On the right side the two Mauthner neurons are magnified and the position of two sections (70 µm thickness) are illustrated with dotted lines. (**b**,**c**) Positive ion MS images. (**b**) MS image of PC (36:1) [M+H]^+^ visualizing the axon arrangement before the point where the axons cross the midline. Axons are labeled with green arrows and a schematic illustration of the axon region is shown on the right side of the MS image. (**c**) MS image of the somata of the two neurons (labeled with green arrows), visualized by the distribution of PC (38:6) [M+H]^+^. (**d**) Fluoresence microscopy of tissue section from transgenic line Tol056 (that expresses GFP in the neuron) was used to conveniently select appropriate sections for subsequent MSI.

After we succeeded in imaging finer details of selected compartments of interest in whole body tissue sections, we further expanded our workflow to be able to characterize an identified large neuron, the Mauthner neuron, within brain sections. In contrast to the sagittal zebrafish sections, sample preparation for brain sections required a more elaborate and careful handling (Fig. 1b). The Mauthner neuron is distinguished by its uniquely large soma, its large-diameter axon and its two major dendrites. Two fixation steps were needed to preserve these structures. Sucrose was used to prevent the cell from swelling during the freezing process. Because this requires time and is done overnight, the tissue first needs to be fixated in paraformaldehyde. Apart from that, euthanization and embedding were performed as described above. Each of the somata of the two Mauthner neurons is located in one hemisphere of the brain and its axon crosses the midline to run down the spinal cord on the side contralateral to the soma (as shown in the scheme in Fig. 3a). To capture this situation, we used coronal slices. This allowed detecting the two axons and localizing their somata. Furthermore, to be able to discriminate steeper gradients in lipid composition, for example between membrane and axoplasma, sufficient spatial resolution was needed and a pixel size of 5 µm was chosen for MS imaging.

To explore if it would be possible to characterize lipid composition at sufficiently high resolution we specifically explored the axon and the soma of the cell. In the axon our method should show no lipids in the axoplasma but high lipid density outside of it. In the soma region, we expected the lipid density to be high. We used a sample thickness of 70 µm. MS imaging of a section that contains the two axons is shown in Fig. 3b. The surrounding tissue in the hindbrain is shown by the distribution of PC (36:1) [M+H]^+^ (Fig. 3b: *m/z* 788.6164). The MS image of the axons shows the absence of lipids within the axoplasma (small dark areas in the MS image, green arrows, in Fig. 3b). This is not only true for PC (36:1), no other lipids were detected in this area. The findings thus do not only demonstrate the high resolution, but also the adequacy of our sample preparation protocol. Had this not conserved the distribution of lipids in the section, the lipid gradient would not have been conserved resulting in lipid signals within the axoplasmatic regions. In a second section, we were able to capture the two somata (in one plane) by MS imaging (Fig. 3c). They are clearly visualized by the distribution of the lipid PC (38:6) [M+H]^+^ (*m/z* 806.5694). It should be noted, that the use of the transgenic line Tol 056 greatly aided in selecting appropriate sections that show the axon or specific areas around the soma using fluorescence microscopy as illustrated by Fig. 3d. This allowed us to specifically analyze the lipid composition in well-defined substructures of a single neuron.

In the past the Mauthner neuron cell could profitably be used to demonstrate the effect of e.g. bisphenols on key neuronal functions^24^. This relies on the size of the neuron, its remarkable accessibility for intracellular electrophysiological recording and its role as an integrator of information from all sensory systems. Many chemical stressors are lipophilic. To test if they can affect the lipid composition of neurons, MS imaging of the Mauthner neuron would thus allow exploring the chemical effect on lipid composition in a single neuron of the vertebrate central nervous system. Moreover, the effects of any such change, once detected with MS imaging, could directly be correlated with electrophysiology to characterize the direct functional consequences.

### Lipid distribution in whole body sections of *D.magna*

Besides *D. rerio*, we also aimed at establishing a methodological approach for analyzing histological effects in another aquatic model organism—the waterflea *Daphnia*. In comparison to *D. rerio* as an important vertebrate model system, *D. magna* is a common invertebrate model organism for freshwater ecosystems and one of the most frequently used ones in aquatic toxicology^20^. Here we present a workflow suitable for the analysis of lipid distribution in cross-sections of non-preserved *D. magna* (Fig. 1c). *D. magna* are small in size (about 1–2 mm) and have a carapace consisting of two opposing integuments that encapsulate the body^25^. The carapace of *D. magna* consists of amorphous calcium carbonate which contributes to its stability^31^. During cryosectioning it often fractures which results in a disruption of body integrity. To overcome this challenge, a series of embedding methods including CMC (3–5%) and gelatin (2–10%) were evaluated. While the anatomical structure was severely damaged using CMC, embedding whole body *D. magna* in 8% gelatine provided the overall best results. Lower concentrations resulted in damaged tissue sections, while higher concentrations resulted in significant reduction of analyte signals due to ion suppression effects. An important practical consideration is that the body cavity within the carapace and appendices of the animal should be filled with the embedding medium in order to keep the fragile thoracic legs from fractioning during cryosectioning. In order to achieve this, the animals were left to swim in the gelatin solution to fill all cavities. Following this sample preparation procedure, we could obtain sections of 18 µm thickness maintaining tissue integrity.

**Figure 4:**
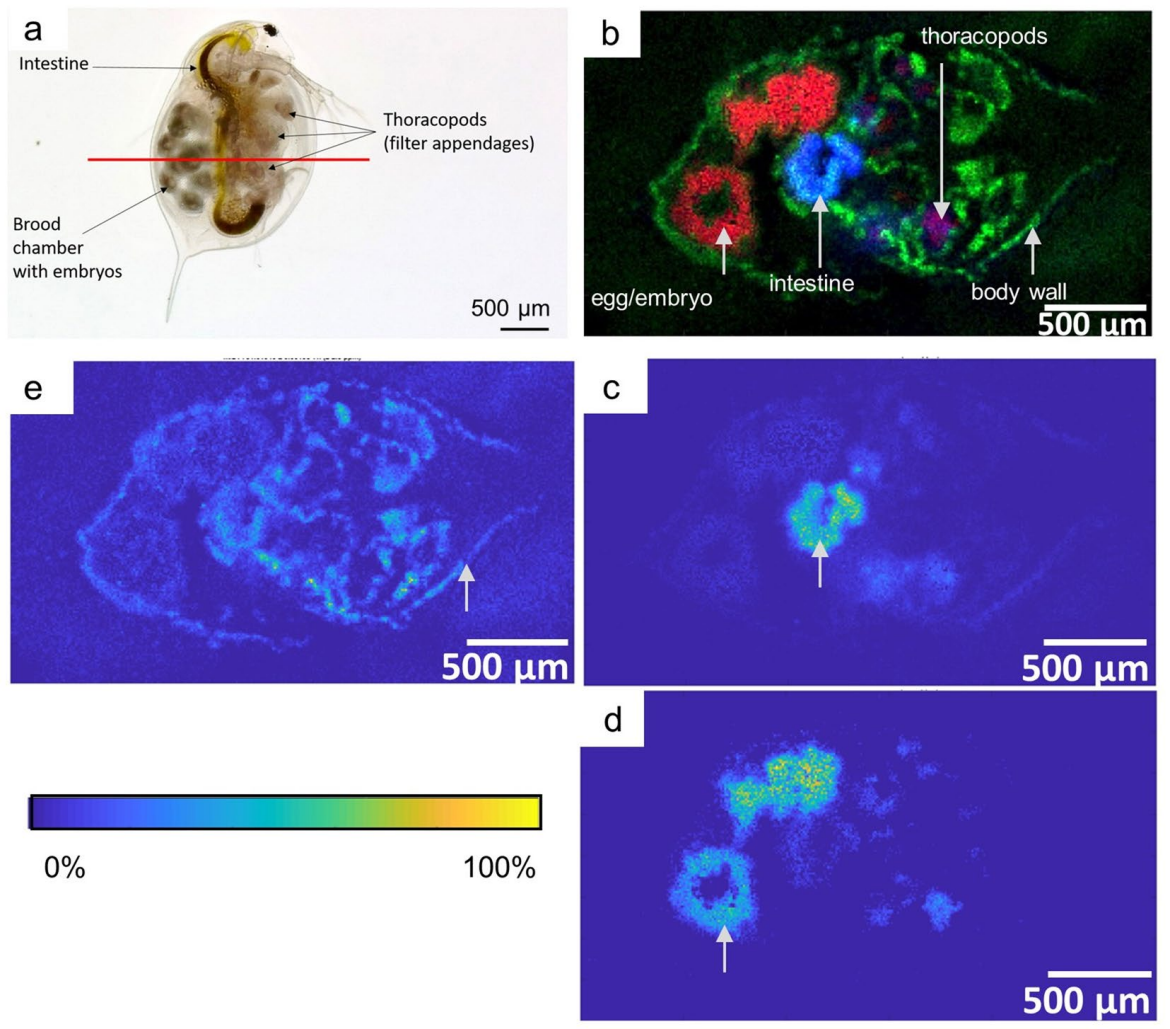
MALDI MSI visualizing the lipid distribution in *D. magna*. (**a**) Whole body image of *D. magna*. (**b**) RGB Overlay of PC (36:0) [M+Na]^+^ (blue colored), HexCer (41:1;O_2_) [M+2Na-H]^+^ (red colored) and SM (38:1;O_2_) [M+Na]^+^ (green colored) visualizing different anatomical regions (thoracic legs, egg/embryo, intestine, body wall). (**b**–**d**) Positive ion MS images. (**c**) Ion image of PC (36:0) [M+Na]^+^, highlighting the intestine region (labeled by a white arrow). (**d**) Ion image of HexCer (41:1;O_2_) [M+2N-H]^+^, highlighting the lipid distribution within the egg/embryo (labeled by a white arrow). (**e**) Ion image of SM (38:1;O_2_) [M+Na]^+^, showing the lipid distribution in the surrounding tissue including body wall (labeled by a white arrow) and thoracic legs.

The anatomical structure of whole *D. magna* is shown in Fig. 4a with the cutting plane indicated by a red line. MS images were acquired at 10 µm pixel size (280 pixel × 260 pixel) in positive ion mode in the mass range of *m/z* 600–1000. The intestine of *D. magna* could be visualized by the distribution of PC (36:0) [M+Na]^+^ (*m/z* 812.6140) (Fig. 4c). In contrast, structures characterized as embryos and eggs could be visualized by the distribution of the glycosylceramide HexCer (41:1;O_2_) [M+2Na-H]^+^ (*m/z* 842.6456) (Fig. 4d). Surrounding structures like the body wall lining the carapace and parts of the thoracic legs could be visualized by the distribution of a sphingomyelin SM (38:1;O_2_) [M+Na]^+^ (*m/z* 781.6194) (Fig. 4e). An RGB overlay of the three lipids within this section is shown in Fig. 4b with HexCer (41:1;O_2_) [M+2Na-H]^+^ colored in red, SM (38:1;O_2_) [M+Na]^+^ colored in green and PC (36:0) [M+Na]^+^ colored in blue. This combined image visualizes anatomical features such as the intestine, integuments and the thoracic legs as well as embryos within the brood pouch in a single measurement.

Analyzing the lipid distribution of daphnids can be of interest regarding the impact assessment of environmental pollutants on the physiology of the organism. For instance, a recent study by Seyoum et al.^42^ was able to show that perfluorinated alkyl compounds impact the lipid metabolism which is discussed as a possible reason of disorders in fecundity. Further, Scanlan et al.^43^ were able to show in a NMR-based lipidomic profiling study that various toxicants such as octabromodiphenyl ether or tetrabromophthalate influence the glycosphingolipid biosynthesis. MALDI MSI offers the possibility to map and thereby to visualize the areas of impact of toxicants directly within a tissue section adding another perspective for understanding the mechanism of action of substances of interest. The gut of *Daphnia* as well as the thoracopods are one of the first contact zones with pollutants. Studies already showed the impact pollutants can have on these compartments. Regarding the gut wall, Heinlaan et al.^44^ evidenced changes in *Daphnia* after exposure to copper oxide nanoparticles with transmission electron microscopy (TEM). This is especially interesting given the fact that alterations of cell walls are most likely associated with changes in lipid distribution. The brood pouch containing the embryos might also be a very interesting target for investigating possible effects of pollutants. Here, studies focused on embryonic development^45^ or allocation of lipids from adult animals to neonates upon different toxicological perturbation factors. The analysis of lipid compositions was for example performed by mass spectrometry after lipid extraction of homogenized samples^46^. Our presented workflow has the advantage that the spatial distribution of targeted molecules can be visualized. This advancement in methodology can enable the assessment or detection of accumulation sites and their effect on an organism.

The current study focuses on lipids as target compounds. However, the obtained tissue sections could be readily used for analysis of other compound classes by adjusting experimental parameters such as matrix selection, polarity and mass range. In order to demonstrate this, an example of a *Daphnia magna* section (prepared with DHB matrix) analyzed in positive ion mode in the *m/z* range 150–600 and *m/z* range 600-1800, respectively, is shown in supplementary information Figure S5. This data shows two metabolites in the intestine of *Daphnia magna*: the dipeptide l-arginyl-l-glutamic acid and the chlorophyll degradation product pheophytin a which supposedly originates from ingested algea. A number of additional candidates have been detected in these preliminary measurements, but were not identified yet. This example shows the potential to expand our workflow—both for *D. magna* and for *D. rerio*—to a wider range of metabolites. Expanding the workflow further to proteins might be a different matter. In this case the critical step is applying the trypsin solution without inducing delocalization of tryptic peptides, as discussed in detail in Huber et al.^47^. This application process might have to be optimized significantly for the fish sections. Adopting it to *D. magna* might prove to be even more challenging due to the more heterogeneous structure and lower level of integrity of these sections.

## Conclusion

In this study we present MALDI MSI workflows that allow the detailed lipid analysis in two important aquatic model organisms, *D. rerio* and *D. magna*. A crucial step to reach this goal was the development of appropriate sample preparation protocols that conserved tissue integrity without affecting ionization efficiency. In *D. rerio* we were able to obtain detailed and high resolution lipid distributions in crucial tissues. Furthermore, we extended the approach to allow the mapping of lipids in a single neuron. For *D. magna*, we were able for the first time to show a suitable sample preparation workflow enabling the application of MALDI MSI on whole body sections. Summarizing, the presented workflow enables the analysis of lipid distribution in different anatomical compartments in *D. rerio* and *D. magna* and is thus a promising tool for future studies focusing on the response of these two important aquatic organisms to water-soluble and particulate pollutants, such as microplastics.

## Methods

### Experimental animals

Adult zebrafish (*D. rerio*) lines used in this study were of the Bt line (wild type) and Casper-Tol-056 line (mutant type). The Casper-Tol-056 line was generated by crossing the pigmentless casper [mitfa^w2/w2^;mpv17^a9/a9^]^48^ strain with the Tol-056 enhancer^49^ trap line, in which the Mauthner neurons, among others, express GFP. All fish used in this study were kept at the Chair of Animal Physiology at the University of Bayreuth. Juvenile fish were raised at 28 °C on a 12:12 h light/dark photoperiod in E3-medium (5 mM NaCl, 0.17 mM KCl, 0.33 mM CaCl_2_, 0.33 mM MgSO_4_ × 7H_2_O, 10^–5^% Methylene Blue in dH_2_O). Adult fish were kept in groups of about 15 animals in commercial fish tanks (Stand-alone unit V60, Aqua Schwarz; Göttingen, Germany; size: 137 × 60 × 231 cm (width, depth, height)) at 28 °C. Water quality parameters were controlled daily (salt content: 0.1 g/L; pH 7.2; water conductivity: 400 µS). Adult fish were fed with commercial fish food (Tetramin: Tetra GmbH; Melle, Germany). Experiments were conducted with adult animals. Animal care procedures and experimental procedures were in accordance with all relevant guidelines and regulations of the German animal protection law and explicitly approved by the animal welfare commission and the animal welfare officer of the University of Bayreuth and by state councils (Regierung von Unterfranken, Würzburg, Germany). The study is reported according to the ARRIVE guidelines^50^.

Adult female *D. magna* (clone: K_3_4J) were cultivated in a climate chamber (20 °C + 1 °C) with a 15:9 h light/dark photoperiod at the Chair of Animal Ecology I at the University of Bayreuth. The daphnids were held on artificial M4 medium^51^ and were fed daily with the green algae *Acutodesmus obliguus*.

### Sample preparation for cryosectioning

Euthanasia of all experimental fish was applied by an overdose of the anaesthetic agent 2-phenoxyethanol. Sectioning of embedded *D. rerio* and *D. magna* samples were performed on a cryomicrotome (CM 3050 S cryostat, Leica Microsystems; Nussloch, Germany). For the wild-type *D. rerio*, 20 µm sagittal sections were prepared at a chamber temperature of − 15 °C. Coronal brain sections of Casper-Tol-056 *D. rerio* were prepared at − 17 °C and 70 µm thickness. Whole-body sections of *D. magna* were prepared at − 27 °C chamber temperature and a thickness of 18 µm.

#### Preparation of sections from wild-type *D. rerio*

Animals were dissected cranial to the anal fin and immediately embedded in 3% carboxymethyl cellulose (CMC; (Sigma-Aldrich, Taufkirchen, Germany)). The embedding medium was prepared by dissolving 750 mg CMC (Sigma-Aldrich, Taufkirche, Germany) in pre-warmed 25 mL Milli-Q water and subsequently cooled to room temperature for further use. For embedding, the cryomold (Science Services, Munich, Germany) was filled with a thin uniform layer of embedding medium and stored at − 20 °C. The prepared *D. rerio* was transferred onto the frozen CMC layer, completely coated by additional CMC medium and immediately stored at − 80 °C until cryosectioning.

#### Preparation of brain sections from the Casper-Tol-056 line

The skull of the fish was opened and the head placed overnight in 4% paraformaldehyde (PFA) in PBS. On the next day the brain was extracted from the scull and transferred to 30% sucrose (Sigma-Aldrich, Taufkirche, Germany), diluted in 4% PFA in PBS. After overnight incubation, the brain was embedded in 3% CMC as it was described before and stored at − 80 °C until cryosectioning.

#### Preparation of *D. magna* sections:

Gelatin embedding solution was prepared by dissolving 800 mg gelatin powder (VWR, Darmstadt, Germany) in 10 mL ultrapure water and the suspension was heated to 55 °C to dissolve the gelatin. The embedding medium was poured in a cryomold and given time to cool down to approximately room temperature. After cooling, a daphnid was placed in a glass bowl filled with embedding medium for approximately 1 minute so that the movement of the thoracic legs spread the embedding medium within the carapace and appendices of *D. magna*. Afterwards, the individual was placed in a cryomold filled with embedding medium and transferred to a brass plate cooled with dry ice. During the freezing process the daphnids were aligned with tweezers. Frozen samples were stored at − 80 °C until cryosectioning.

### Matrix application

Prior to matrix application, *D. rerio* and *D. magna* sections were placed in a desiccator for 1 h to avoid condensation on the sample surface. Matrix application was carried out using a semi-automatic pneumatic sprayer system built in house. All sections were coated with 4-nitroanilin matrix (pNA, ≥ 99%, Sigma Aldrich Chemie, Taufkirche, Germany) at 5 mg/mL in 3:1 acetone/water.

### MALDI MSI measurements

MALDI MSI measurements were performed on a QExactive™ HF Hybrid-Quadrupole-Orbitrap mass spectrometer (Thermo Fisher Scientific GmbH, Bremen, Germany) coupled to an AP-SMALDI5 source (TransMIT GmbH, Gießen, Germany) equipped with a λ = 343 nm solid state laser operating at a repetition rate of 100 Hz. Measurements were carried out in positive ion mode with a mass range of *m/z* 600–1000 with one scanning event per pixel at a mass resolution of 240 k @ *m/z* 200 full width at half maximum (FWHM). All measurements were performed with a fixed C-trap injection time of 500 ms. Step sizes were set to 25 µm for the sagittal wild-type zebrafish line sections, 10 µm for *D. magna* sections and 5 µm for the coronal Casper-Tol-056 *D. rerio* line sections. Tentatively identification of lipids from *D. rerio* sections was based on online data base search and on tissue MS/MS of lipids with a precursor isolation window width of ± 0.2 m*/z*. Tentatively identification of lipids in *D. magna* sections was based on online data base search^52^.

### Data processing and image generation

Conversion of proprietary Thermo RAW files to imzML was performed using the java based open access software ‘*jimzML’ Converter*^53^*.* Ion images and RGB composite images were generated in the open source software *MSiReader Version 1.0*.^54^. Images were generated using a bin width ± 2.5 ppm. Mass deviations across imaging datasets are given as the root mean square error (RMSE) of the ∆m values in ppm of each individual spectrum containing the targeted analyte.

### H&E staining

*Danio rerio* sections were used for H&E staining after MALDI MSI experiments. Prior to staining, the matrix layer was removed with acetone. The sections were then rehydrated with decreasing ethanol concentrations (2 min in 100%, 70%, 40%), rinsed in 100% distilled water and stained with Mayer’s Hematoxylin Solution for 12 min, before sections were submerged in tab water and rinsed again in distilled water. 0.5% acidified Eosin Y was used for counterstaining. Stained sections were fixated in xylol and mounted with Eukitt mounting medium and coverslips.

## Supplementary Information

Supplementary Information.
