# Supplementary material for: MALDI mass spectrometry imaging workflow for the aquatic model organisms Danio rerio and Daphnia magna
Source: Sci Rep. 2022 May 4;12:7288. doi: 10.1038/s41598-022-09659-y (PMC9068711; doi:10.1038/s41598-022-09659-y)
Supplement: Supplementary file 1 — Supplementary Information. [file 41598_2022_9659_MOESM1_ESM.pdf]

MALDI mass spectrometry imaging workflow for the aquatic model organisms *Danio rerio* and *Daphnia magna*

Elisabeth Schirmer, Sven Ritschar, Matthias Ochs, Christian Laforsch, Stefan Schuster & Andreas Römpp

Supplementary information

Supplementary Table S1. RMS-Errors [ppm] of preliminary identified lipid adducts shown including theoretical *m/z* values, the number of spectra ($n_{Spectrum}$) analyzed for RMS-Error calculation & figure-references.

***m/z* theoretical**	**Lipid-Adduct**	**RMS-Error [ppm]**	**RMS-Error** ($n_{Spectrum}$)	**Figure-Reference**
720.5902	PC O-32:0[M+H]$^+$	1.31	24780	Fig. 2c
744.494	PC 30:0[M+K]$^+$	1.25	26093	Fig. 2d
720.5902	PC O-32:0[M+H]$^+$	0.87	33692	Fig. 2g
812.593	PC O-36:1[M+K]$^+$	1.33	22349	Fig. 2h
834.6007	PC 40:6 [M+H]$^+$	1	57107	Fig. 2i
788.6164	PC 36:1[M+H]$^+$	0.82	42070	Fig. 3b
806.5694	PC 38:6 [M+H]$^+$	1.82	33086	Fig. 3c
812.614	PC 36:0 [M+Na]$^+$	1.25	20868	Fig. 4c
842.6456	HexCer 41:1;O_2 [M+2Na-H]$^+$	0.88	5189	Fig. 4d
781.6194	SM 38:1;O_2 [M+Na]$^+$	0.65	31188	Fig. 4e
812.614	PC 36:0 [M+Na]$^+$	1.16	6717	Supplementary Fig. 4b
842.6456	HexCer 41:1;O_2 [M+2Na-H]$^+$	0.78	20893	Supplementary Fig. 4c
781.6194	SM 38:1;O_2 [M+Na]$^+$	1.14	21744	Supplementary Fig. 4d

Supplementary Figure S1. Selected single spectra of *m/z* values (boxed) plotted in Fig. 2. Mass deviations (Δppm) and mass resolutions are provided. Single mass spectrum of *m/z* corresponding to **(a)** Fig. 2c, **(b)** Fig. 2d, **(c)** Fig. 2g, **(d)** Fig. 2h and **(e)** Fig.2i. Spectra were acquired with R = 200.000 at *m/z* 200 (FWHM).

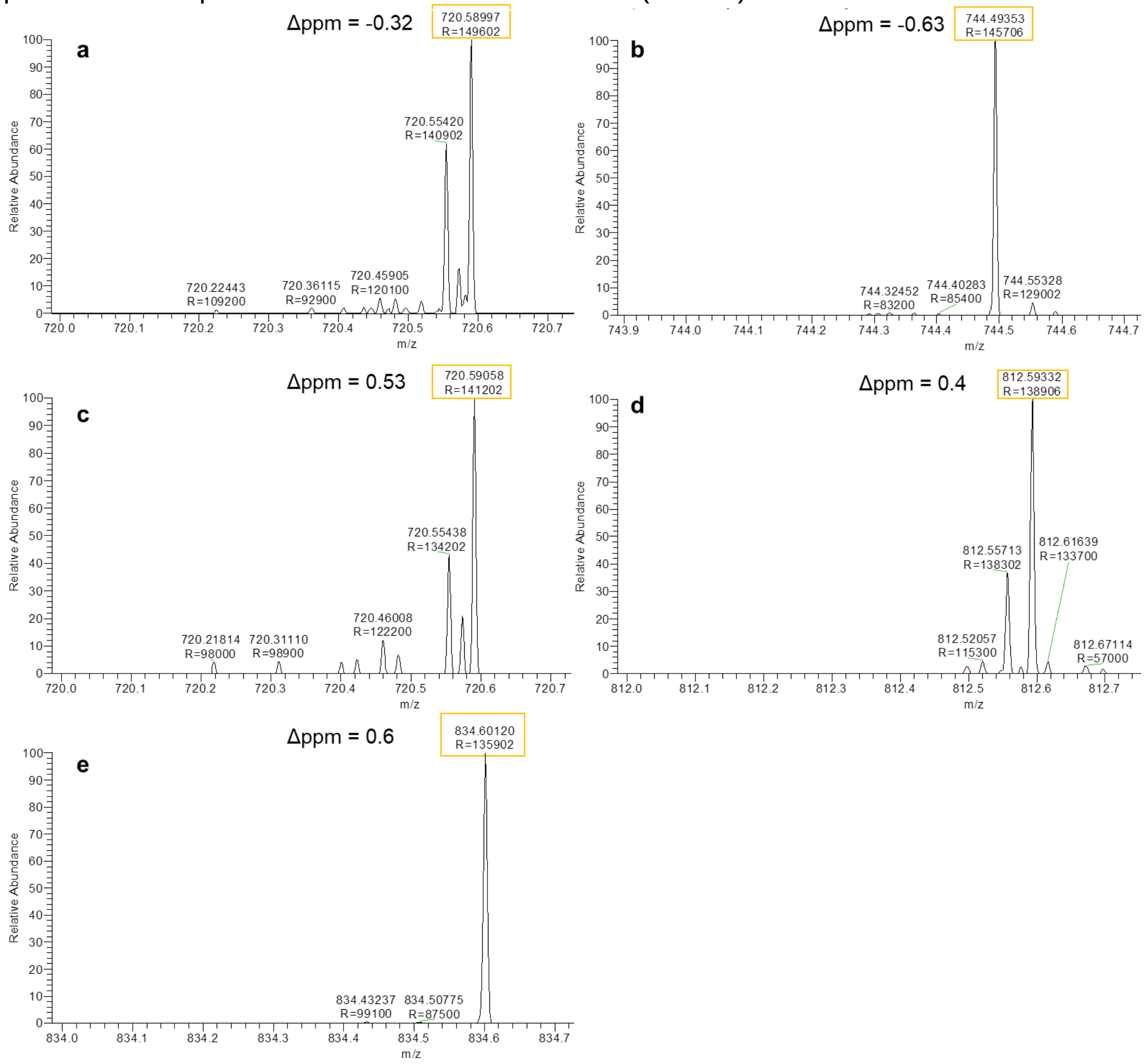

Supplementary Figure S2. Selected single spectra of *m/z* values (boxed) plotted in Fig. 3. Mass deviations (Δppm) and mass resolutions are provided. Single mass spectrum of *m/z* corresponding to **(a)** Fig. 3b and **(b)** Fig. 3c. Spectra were acquired with R = 200.000 at *m/z* 200 (FWHM).

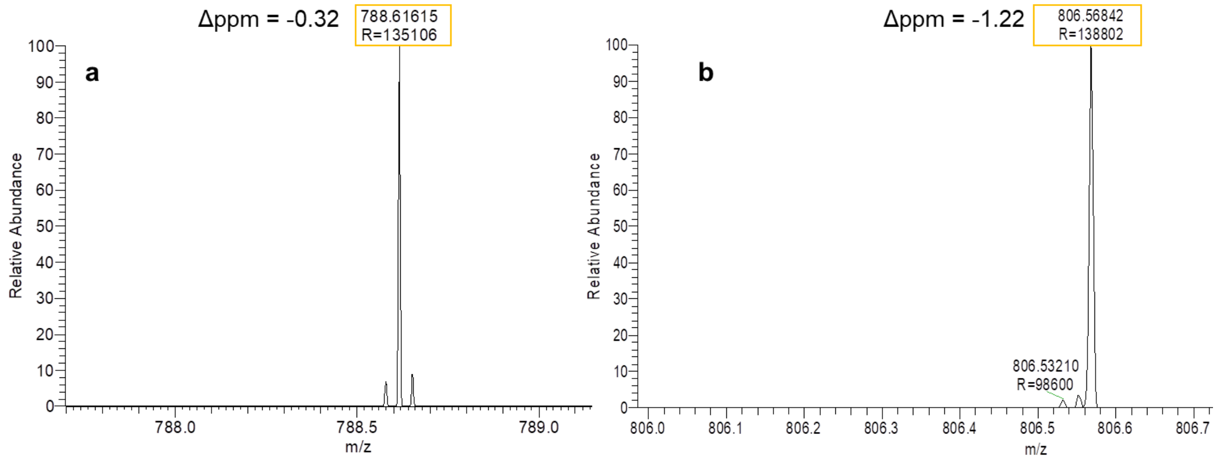

Supplementary Figure S3. Selected single spectra of *m/z* values (boxed) plotted in Fig. 4. Mass deviations (Δppm) and mass resolutions are provided. Single mass spectrum of *m/z* corresponding to **(a)** Fig. 4c, **(b)** Fig. 4d and **(c)** Fig. 4e. Spectra were acquired with R = 200.000 at *m/z* 200 (FWHM).

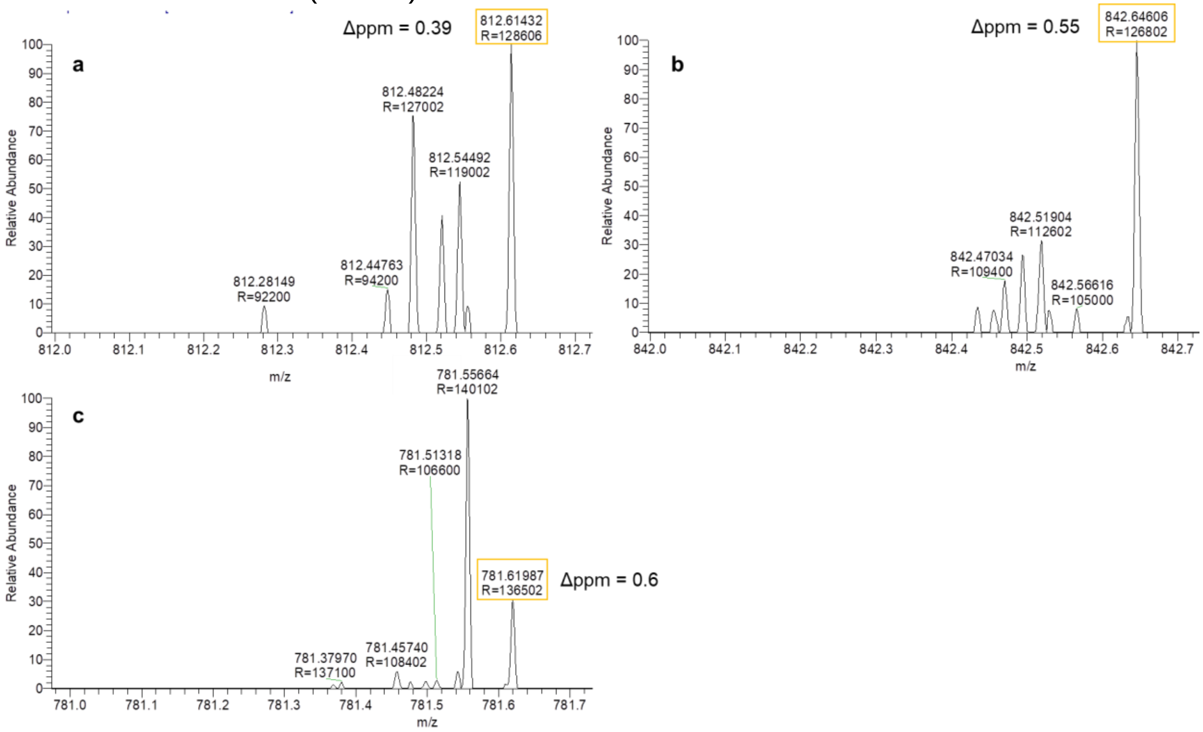

Supplementary Figure S4. *Replicate MALDI MSI measurement* visualizing the lipid distribution in neighboring section of *D. magna*. **(a)** RGB Overlay of **PC (36:0) [M+Na]$^+$**, **HexCer (41:1;O_2) [M+2Na-H]$^+$** and **SM (38:1;O_2) [M+Na]$^+$** visualizing different anatomical regions (thoracic legs, egg/embryo, intestine, body wall). **(b-d)** shows positive-ion MS images. **(b)** Ion image of PC (36:0) [M+Na]$^+$ highlighting the intestine region. **(c)** Ion image of HexCer (41:1;O_2) [M+2N-H]$^+$ highlighting the lipid distribution within the egg/embryo. (**d**) Ion image of SM (38:1;O_2) [M+Na]$^+$ showing the lipid distribution in the surrounding tissue, including body wall and thoracic legs. **(e)** Optical image of the analyzed *D. magna coronal section*.

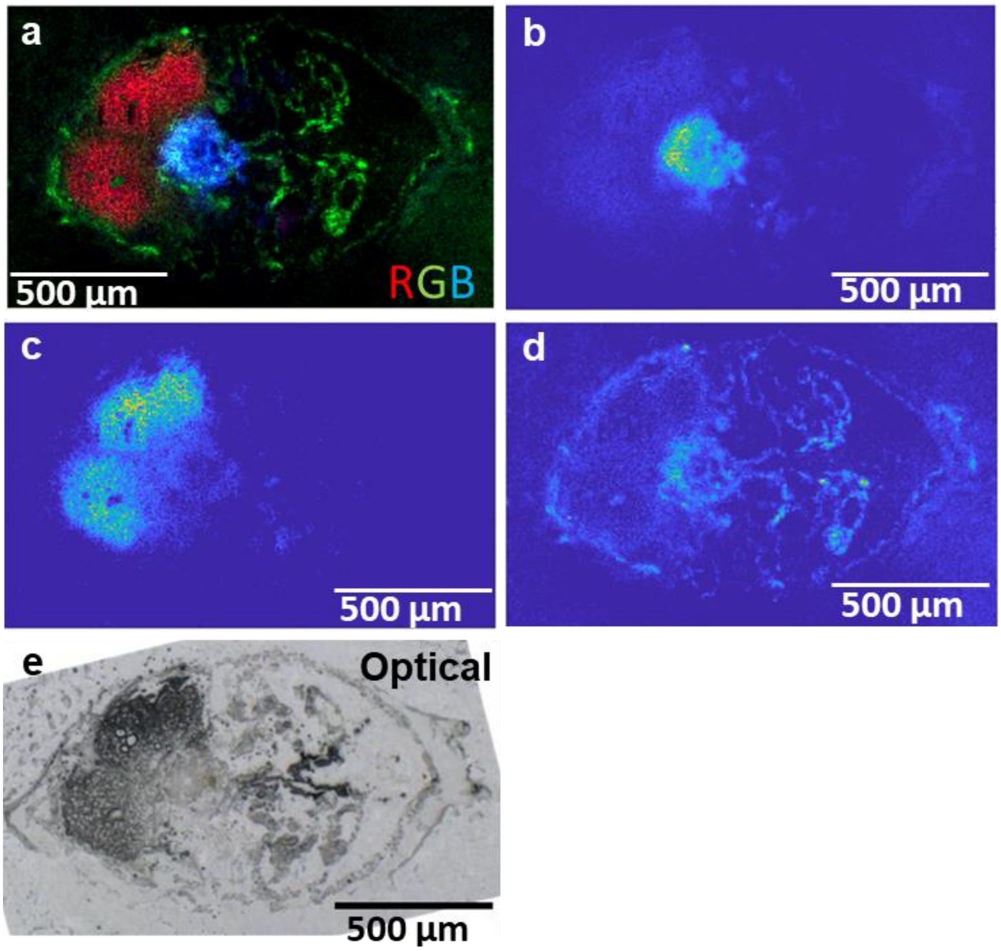

Supplementary Figure S5. MALDI MSI measurement visualizing the distribution of representatives of two other compound classes (dipeptide & chlorophyll). (a) Optical image of the analyzed *D. magna* coronal section. **(b)** Ion image of L-arginyl-L-glutamic acid $[M+H]^+$ predominantly located in the intestine region. **(c)** Ion image of pheophytin a $[M+H]^+$ detected in the gut.

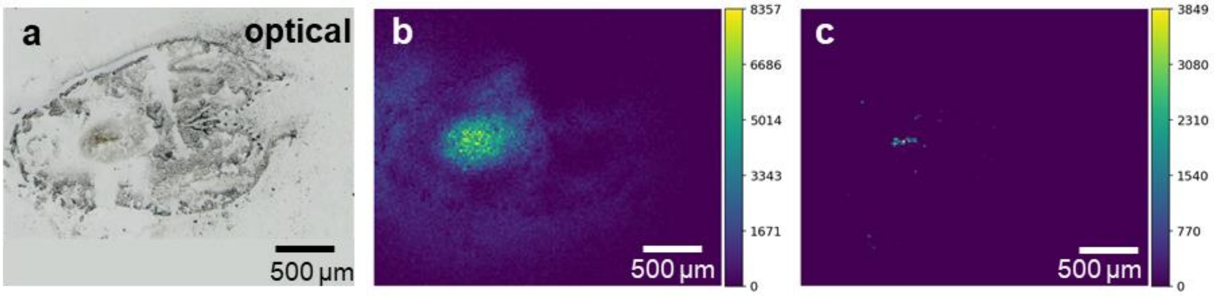

The metabolites shown in Figure S5 were tentatively annotated via Metaspace[1] using ChEBI as database and a false-discovery-rate ≤ 10 %[2].

Supplementary References

1 Metaspace. The platform for metabolite annotation of imaging mass spectrometry data, <https://metaspace2020.eu/>

2 Palmer, A. *et al.* FDR-controlled metabolite annotation for high-resolution imaging mass spectrometry. *Nat Methods* **14**, 57-60, doi:10.1038/nmeth.4072 (2017).
